# Different Intestinal Microbial Profile in Over-Weight and Obese Subjects Consuming a Diet with Low Content of Fiber and Antioxidants

**DOI:** 10.3390/nu9060551

**Published:** 2017-05-27

**Authors:** Tania Fernández-Navarro, Nuria Salazar, Isabel Gutiérrez-Díaz, Clara G. de los Reyes-Gavilán, Miguel Gueimonde, Sonia González

**Affiliations:** 1 Area of Physiology, Department of Functional Biology, University of Oviedo, 33006 Asturias, Spain; tfnavarro214@gmail.com (T.F.-N.); igutidiaz@gmail.com (I.G.-D.); 2 Department of Microbiology and Biochemistry of Dairy Products, Instituto de Productos Lácteos de Asturias, Consejo Superior de Investigaciones Científicas (IPLA-CSIC), Paseo Río Linares s/n, Villaviciosa, 33300 Asturias, Spain; greyes_gavilan@ipla.csic.es (C.G.d.l.R.-G.); mgueimonde@ipla.csic.es (M.G.)

**Keywords:** oxidative stress, microbiota, obesity, antioxidant, western diet

## Abstract

Obesity has been related to an increased risk of multiple diseases in which oxidative stress and inflammation play a role. Gut microbiota has emerged as a mediator in this interaction, providing new mechanistic insights at the interface between fat metabolism dysregulation and obesity development. Our aim was to analyze the interrelationship among obesity, diet, oxidative stress, inflammation and the intestinal microbiota in 68 healthy adults (29.4% normal-weight). Diet was assessed through a food frequency questionnaire and converted into nutrients and dietary compounds using food composition tables. The intestinal microbiota was assessed by quantitative PCR, fecal short chain fatty acids by gas chromatography and serum biomarkers by standard protocols. Higher levels of malondialdehyde (MDA), C reactive protein (CRP), serum leptin, glucose, fat percentage and the intestinal *Lactobacillus* group were found in the obese people. Cluster analysis of body mass index, fat mass, glucose, LDL/HDL ratio, leptin, MDA and CRP classified the subjects into two groups. The levels of the intestinal *Bacteroides-Prevotella-Porphyromonas* group were lower in the cluster and linked to a higher pro-oxidant and pro-inflammatory status, whose individuals also had lower intake of fruits, dried fruits, and fish. These results could be useful for designing strategies targeted to obesity prevention.

## 1. Introduction

The prevalence of obesity is growing worldwide, with nearly half a billion of the world’s population considered to be overweight. Obesity, defined by the World Health Organization (WHO) as an “abnormal or excessive fat accumulation”, has been associated with an increasing risk of multiple diseases, characterized by changes in the oxidative/antioxidant balance and the presence of subclinical inflammation [1,2,3,4], including diabetes, metabolic syndrome, hypertension, dyslipidemia and cardiovascular disease among others [5]. Thanks to the great efforts made in obesity research during the last decades, several genetic, environmental and lifestyle-related factors have been identified as etiological risk elements for this condition. However, there are still factors in this equation, such as the gut microbiota, whose contribution remains to be fully elucidated. It has been proposed that the reduction of carbohydrates accessible to gut microbes from fiber-containing foodstuffs may result in a long-term reduction of microbiota diversity and in the appearance of “unhealthy” microbiomes [6,7,8]. Although it has not been possible to establish the directionality of this relationship, the gradual increase in energy intake, together with the dramatic change in the proportion of macronutrients of the Western diet, seems to be linked with the increase of the obesity prevalence [9] mediated by changes in intestinal microbial populations.

In contrast, the content in bioactive compounds of the Mediterranean Diet has been linked with the prevention of obesity and metabolic syndrome, by means of restoring the intestinal microbial balance of these patients [10,11]. An increase of the intestinal *Firmicutes/Bacteroidetes* ratio has been reported in several studies with obese humans [12], in obese leptin deficient ob/ob mice [13] and in wild-type animals receiving Western diets [14]. The benefits of dietary fiber in host health have been reported to be partly mediated by physiological effects linked to the formation in the colon of fecal short chain fatty acids (SCFA) produced by the microbial fermentation of complex carbohydrates. In this regard, it is known that the major SCFA synthesized by the colonic microbiota (acetic, propionic, and butyric) play a key role in regulating host energy balance in extra-intestinal organs, such as the liver and adipose tissue [15,16]. In an apparently contradictory way, high levels of fecal SCFA have been frequently found in obese subjects [17]. The specific mechanisms explaining the higher levels of fecal SCFA in obesity still remain a matter of debate and different hypotheses have been proposed, including: an increase on colonic fermentation due to the higher dietary intake; a higher capacity for energy harvest by the modification of the intestinal microbial metabolic profile linked to obesity [18,19]; and the reduction of the in vivo fluxes of SCFA from the intestinal lumen to other host organs, with accumulation in the colonic lumen [20].

The currently available evidences highlight the complex network of physiological mechanisms underlying obesity [21]. In this scenario, the intestinal microbiota may provide new mechanistic insights at the interface between fat metabolism dysregulation and obesity development. To the best of our knowledge, there are no currently available interdisciplinary studies addressing the interrelationships among obesity, diet, oxidative stress, inflammation and gut microbiota. However, a better understanding of these interactions could be useful for the development of new approaches for preventing and controlling obesity. Therefore, our aim was to determine differences in the serum concentration of malondialdehyde (MDA), glucose, lipid profile, and C reactive protein (CRP), according to the grade of obesity, to describe the gut microbial composition linked with this pathology and to analyze the role of the diet in the possible associations among these parameters.

## 2. Subjects and Methods

### 2.1. Participants

This cross-sectional study is part of a research into diet and gut microbiota in different population groups. The study sample comprised 68 adult volunteers 27 men and 41 women, aged from 19 to 67 years (mean ± SD, 52.4 ± 11.2). Participants were recruited in Asturias Region (Northern Spain) between the years 2009 and 2015 among subjects attending a program of the University of Oviedo for people older than 50 years, as well as among individuals participating in a study on nutritional habits from the Alimerka Foundation. In a personal interview, volunteers were informed of the objectives of the study and those deciding to participate gave their fully informed written consent. Then, personal appointments were made to collect the dietary information. Subjects were initially classified according to their Body Mass Index (BMI) [22]. From the initial sample four subjects were excluded from cluster analysis because no data were recorded for some of the parameters introduced as variables in our study. Moreover, three subjects had missing values for fecal microbiota and, therefore, were not included for further analyses. The following inclusion criteria were used: not being diagnosed of autoimmune diseases, inflammatory bowel disease or other conditions known to affect the intestinal function, as well as not having undergone medical treatment with oral corticoids, immunosuppressive agents, monoclonal antibodies, antibiotics or immunotherapy or not having consumed any supplement containing probiotics or prebiotics during the previous month. Volunteers diagnosed for diabetes mellitus type II were specifically excluded from the study. Ethical approval for this study was obtained from the Bioethics Committee of CSIC (Consejo Superior de Investigaciones Científicas) and from the Regional Ethics Committee for Clinical Research (“Servicio de Salud del Principado de Asturias nº13/2010”) in compliance with the Declaration of Helsinki of 1964. All experiments were carried out in accordance with approved guidelines and regulations.

### 2.2. Nutritional Assessment

Dietary intake was assessed in a personal interview by means of an annual semi-quantitative food frequency questionnaire (FFQ) method which details 160 items and has been widely used and validated in previous studies [23,24]. The consumption of foods was converted into energy and macronutrients using the nutrient food composition tables developed by the Centro de Enseñanza Superior de Nutrición y Dietética (CESNID) [25]; the intake of monosaccharides (glucose, galactose, fructose) sucrose, starch and digestible polysaccharides and trans fatty acids was converted using the National Nutrient Database for Standard Reference from the United States Department of Agriculture (USDA) [26]. Information about dietary soluble and insoluble fibers was completed from Marlett et al. [27] and phenolic intake was estimated from the Phenol Explorer database [28]. Data about the Oxygen Radical Absorbance Capacity (ORAC) of foods was obtained from the Database from the ORAC of Selected Foods from USDA [29]. This database reports the hydrophilic ORAC, lipophilic ORAC and total ORAC values for 275 foods as μmol of Trolox equivalents (TE)/100 g. For the estimation of these variables in the study sample we used the information contained in our database regarding the daily intake of each food in g/day for all the evaluated subjects. This information has been crossed with the data contained in the ORAC database in order to calculate the intake of ORAC hydrophilic, lipophilic and total in μmol TE for each food and subject. Once this partial information was available, the summary of all the hydrophilic ORAC, lipophilic ORAC and total ORAC obtained per subject was calculated in order to estimate the total antioxidant capacity of the diet by subject.

### 2.3. Anthropometric Measures

At the same time of carrying out the blood extraction, between eight and nine o’clock and after over-night fast, anthropometric measures were taken. Height was registered using a stadiometer with an accuracy of ±1 mm (Año-Sayol, Barcelona, Spain). Subjects stood barefoot, in an upright position and with the head positioned in the Frankfort horizontal plane. Weight was measured on a scale with an accuracy of ±100 g (Seca, Hamburg, Germany). BMI was calculated and stratified according to the Sociedad Española para el Estudio De la Obesidad (SEEDO) [22] criteria: lean–normal weight (≤25.0 kg/m^2^), over-weight (25.0–30.0 kg/m^2^), and obese (≥30.0 kg/m^2^). Body fat percentage was measured by bioelectrical impedance (BIA) with ±1% variation, with subjects in light clothes and in fasted state (Tanita Corporation of America, Inc., Arlington Heights, IL, USA). Basal Metabolic Rate (BMR) was calculated by the Harris-Benedict formula [30].

### 2.4. Blood Biochemical Analyses

Fasting blood samples were drawn by venepuncture after a 12 h fast and collected in separate tubes for serum and plasma. Samples were kept on ice and centrifuged (1000× *g*, 15 min) within 2–4 h after collection. Plasma and serum aliquots were kept at −20 °C until analyses were performed. Plasma glucose, cholesterol, and triglycerides were determined by standard methods. Serum levels of CRP were determined by using a CRP Human Instant ELISA kit (Ebioscience, San Diego, CA, USA), and those of MDA with a colorimetric assay of lipid peroxidation (Byoxytech LPO-586, Oxis International S.A., Paris, France); the within-run coefficient of variation ranged from 1.2% to 3.4%, depending on the concentration of MDA [31].

Serum leptin was measured by a sensitive ELISA test (Human Leptin ELISA Development Kit, 900-K90 PeproTech Inc., Rocky Hill, NJ, USA) according to the manufacturer’s instructions. The detectable concentration range was 63–4000 pg/mL. The intra-assay and interassay coefficients of variation were 5.21% and 5.20%, respectively.

### 2.5. Fecal Collection and Microbiological Analyses

Participants received detailed instructions to collect fecal samples and were provided with a sterile container. Samples were immediately frozen at −20 °C after deposition. For analyses, fecal samples were melted, weighed, diluted 1/10 in sterile PBS, and homogenized in a LabBlender 400 Stomacher (Seward Medical, London, UK) for 4 min; the DNA was extracted using the QIAamp DNA stool mini kit (Qiagen, Hilden, Germany) as previously described [32]. Quantification of different bacterial populations that covered the major bacterial groups present in the gut microbial ecosystem (Table 1) was performed in feces with a 7500 Fast Real-Time PCR System (Applied Biosystems, Foster City, CA, USA) using SYBR Green PCR Master Mix (Applied Biosystems). One microlitre of template fecal DNA (~5 ng) and 0.2 μM of each primer were added to the 25-μL reaction mixture. PCR cycling consisted of an initial cycle of 95 °C 10 min, followed by 40 cycles of 95 °C 15 s, and 1 min at the appropriate primer-pair temperature (Table 1). The number of cells was determined by comparing the Ct values obtained from a standard curve constructed using the pure cultures of appropriate strains that were grown overnight in GAM (Gifu Anaerobic Medium) medium (Nissui Pharmaceutical Co., Tokyo, Japan) under anaerobic conditions (Table 1). The Ct values were plotted as a linear function of the base-10 logarithm of the number of cells calculated by plate counting. Fecal DNA extracts were analysed and the mean quantity per gram of fecal wet weight was calculated.

### 2.6. Short Chain Fatty Acids (SCFA) Analyses

The analysis of SCFA was performed by gas chromatography to determine the concentrations of acetate, propionate and butyrate. Supernatants from 1 mL of the homogenized feces were obtained by centrifugation and filtration as previously indicated [32,33]. A chromatograph 6890N (Agilent Technologies Inc., Palo Alto, CA, USA) connected to a mass spectrometry detector (MS) 5973N (Agilent Technologies) and a flame ionization detector (FID) was used for identification and quantification of SCFA, respectively. Chromatographic conditions and SCFA analyses were carried out essentially as described by Salazar et al. [34].

### 2.7. Statistical Analysis

Statistical analysis was performed using the IBM SPSS program version 22.0 (IBM SPSS, Inc., Chicago, IL, USA). Goodness of fit to the normal distribution was analyzed by means of the Kolmogorov-Smirnov test. When the distribution of variables was skewed, the natural logarithm of each value was used in the statistical test. Overall, categorical variables were summarized with counts and percentages while continuous variables were summarized using means and standard deviations. The chi-squared test and independent samples *t*-test were used for group comparisons where appropriate. Differences in general characteristics, anthropometric, blood parameters, major microbial target and SCFA were assessed in accordance to body mass index classification by means of uni- and multivariate analyses controlling by gender or energy, as appropriate. Also, in order to explore the association between the intestinal microbiota and SCFA with BMI, a linear regression was conducted.

Using the program R (version 3.3.1 for Windows), a cluster analysis using the Ward’s method was performed in order to classify the participants based on the similarity of the different obesity related factors evaluated (BMI, percentage of fat mass, serum glucose, leptin, MDA, CRP and LDL/HDL ratio). This is a hierarchical cluster technique done on the basis of Euclidean distances; therefore, the centers of clusters are grounded on least squares estimation. Differences in the intake of food groups, macronutrients and some dietary components, including antioxidants, were obtained by means of a multivariate analysis controlling for energy intake. The conventional probability value of 0.05 was used in the interpretation of results to indicate statistical significance.

## 3. Results

The general characteristics of the sample classified according to BMI are presented in Table 2.

As was expected, obese subjects had significantly higher basal metabolic rate, percentage of fat mass, serum glucose, leptin, MDA and CRP than normal weight volunteers. Regarding the fecal microbial composition and the microbial metabolic activity (SCFA), the levels of *Lactobacillus* group and acetate concentrations were directly related with the grade of obesity (Table 3).

**Figure 1:**
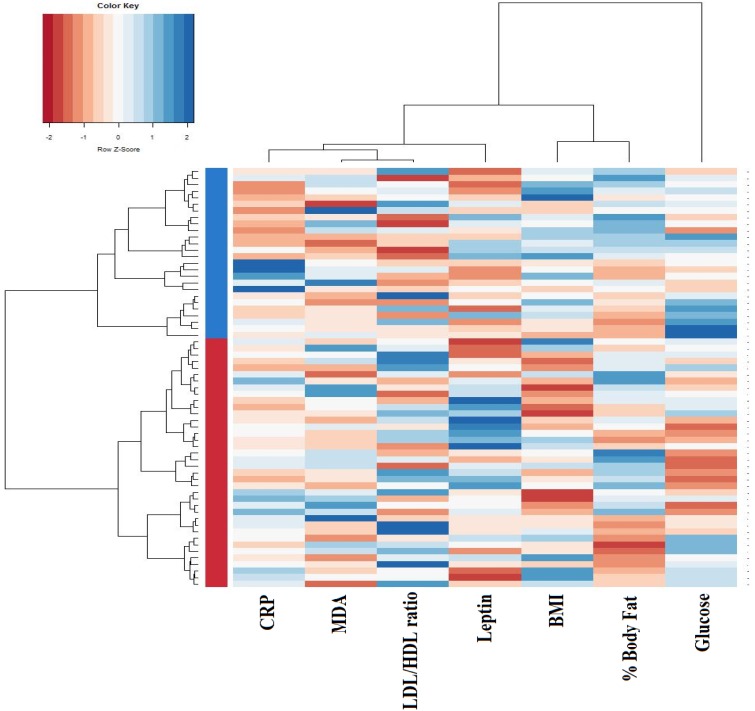
Dendogram clustering based on individual body composition, serum glucose, lipid profile, and oxidative stress biomarkers. The heatmap shows the dendrogram classification for clusters, based on C reactive protein (CRP), malondialdehyde (MDA), LDL/HDL ratio, serum leptin, Body Mass Index (BMI), body fat percentage and serum glucose (columns). Colors in the vertical bar at the left of the heatmap identify Cluster I (red) and Cluster II (blue).

To explore whether the fecal microbiota may be related with the pro-oxidant and pro-inflammatory status frequently linked to obesity, subjects were classified into clusters performed by jointly considering values for BMI, fat mass, serum glucose, LDL/HDL ratio, serum leptin, MDA and CRP. This approach established two independent groups of individuals, thereafter referred to as cluster I (*n* = 38) and cluster II (*n* = 26) (Figure 1).

Cluster II was characterized by higher BMI and body fat together with higher concentration of serum glucose, MDA and CRP. The LDL/HDL ratio and serum leptin, however, did not show significant differences between both clusters (Table 4).

When we compared the microbiota variables between both groups no significant differences were found for most of the microbial groups and SCFA studied, except for acetate, which displayed higher fecal concentration in cluster II, and the *Bacteroides* group whose levels resulted higher in cluster I (Table 5).

**Figure 2:**
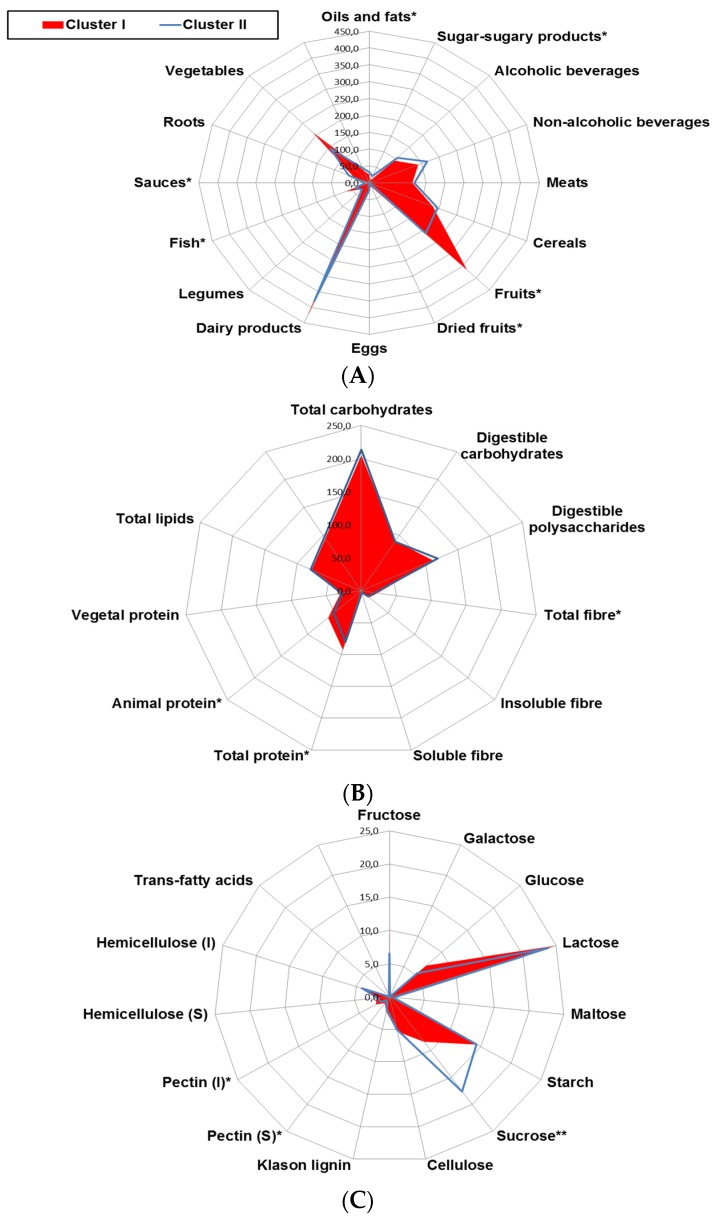
Radar plot representing differences in the daily intake of (**A**) major food groups (g/day); (**B**) macronutrient and fiber (g/day) and (**C**) detailed carbohydrate and fibers (g/day) among clusters. Cluster I (*n* = 38), Cluster II (*n* = 26). Multivariate regression analyses were adjusted by energy intake (Kcal/day). * *p* ≤ 0.05.

With the above information, and in order to evaluate whether differential daily consumption of foods may be related with the pro-oxidant and pro-inflammatory status linked to obesity, food groups and nutrient intake profiles were compared between clusters I and II (Figure 2). Cluster II was characterized by a profile with a higher intake of oils and fats, sweetened foods and sauces and lower intake of fruits, dried fruits, and fish, representative of a Western pattern (Figure 2A). Among nutrients, higher intake of sucrose and lower intake of fiber and total animal protein was observed in individuals from cluster II (Figure 2B,C).

The intake of major antioxidants among clusters was also investigated (Table 6). Subjects from cluster II obtained lower total antioxidant capacity from diet, both for water soluble and fat soluble antioxidant compounds (hydrophilic and lipophilic ORAC, respectively). This group also had lower intake of total carotenoids, vitamin C, total polyphenols and flavonoids than cluster I.

**Figure 3:**
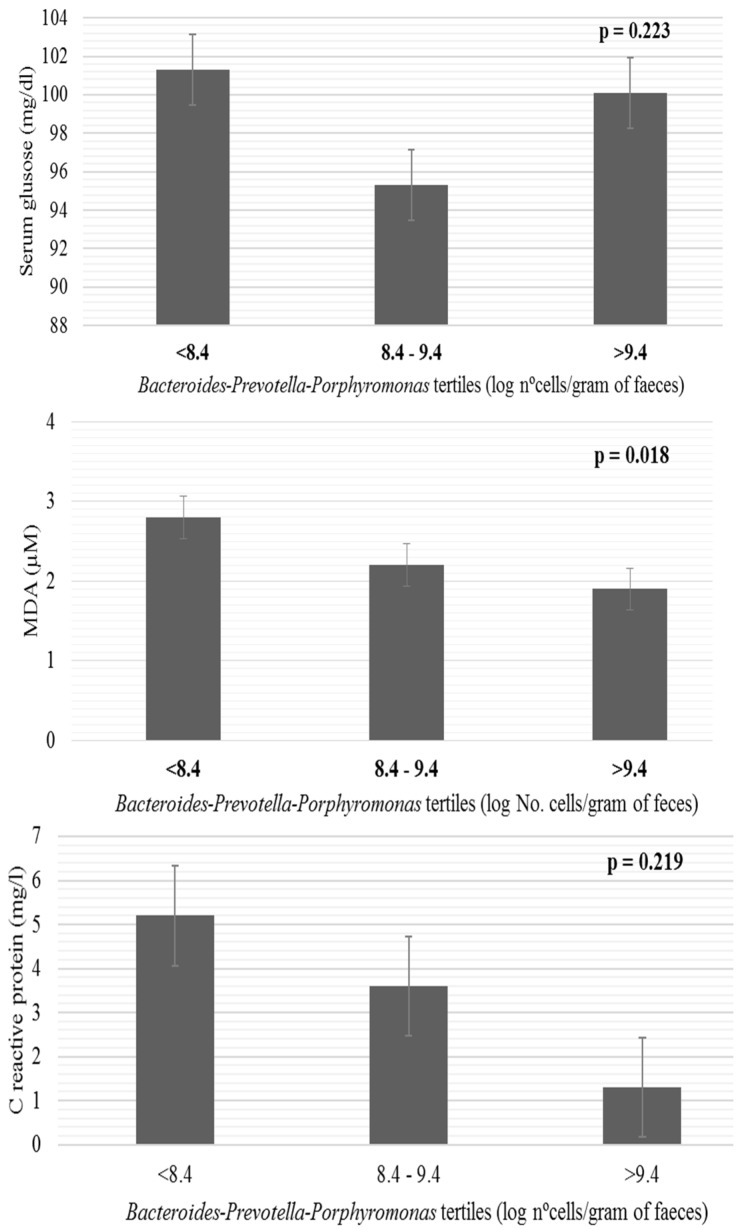
Differences in blood parameters related to obesity according to the levels of *Bacteroides-Prevotella-Porphyromonas* tertiles (log nº cells/gram of feces). Bars represent mean and whiskers standard error derived from univariate analysis adjusted by energy. MDA, malondialdehyde.

In order to elucidate the role of the fecal microbiota in the observed associations, differences in some biomarkers related to obesity according to the tertiles formed with the fecal levels of *Bacteroides-Prevotella-Porphyromonas* (log nº cells/gram of feces) were assessed (Figure 3). Higher serum concentrations of MDA were found across the tertiles of *Bacteroides-Prevotella-Porphyromonas* in relation with the decrease in abundance of this microbial group.

## 4. Discussion

In line with previous evidence from other authors, an association between obesity and higher serum concentrations of leptin [35], MDA [3,36], CRP [3] and glucose was corroborated in our study. However, our work also provides novel insight into the differences found in some intestinal bacterial groups related to BMI and obesity-associated oxidative stress and inflammation in the general population. Furthermore, the identification of different dietary patterns in overweight and obese people, who have a more pro-oxidant and inflammatory status than the normal-weight group, points to the potential interest of designing strategies based on the consideration of the impact of a balanced diet (with high content of antioxidants in comparison with fats and refined sugars) on the microbiota, in order to improve or prevent obesity-associated disorders.

In recent years, hyperleptinemia has also been identified as an independent risk factor for cardiovascular disease and myocardial infarction [37,38] directly linked to inflammation [39] and oxidative stress [3,35]. In the present study, we have found that leptin level increased proportionally with the degree of obesity (Table 2) and is positively correlated with adiposity (Pearson correlation with the percentage of fat mass *r* = 0.590, *p* < 0.001, data not shown), its serum concentration being similar to that previously reported by other authors [40,41]. Higher serum levels of MDA and CRP have been previously reported in obesity and metabolic syndrome [42] and have also linked obesity to alterations in the lipoprotein particles profile as well as to increased lipid peroxidation, during which the MDA is one of the most abundant products formed [43,44,45,46,47]. In this way, our results regarding levels of MDA are in-line with previous findings and the levels of CRP corroborate studies relating to the presence of obesity with chronic low-grade inflammation [48]. In our sample the mean concentrations of MDA and CRP were higher in obese than in overweight and normal-weight groups, however the range observed for MDA was far from the cutoff points associated with increased risk of mortality by different causes [49]. It should be noted in the interpretation of these results that we have excluded people with cancer, autoimmune, or digestive diseases, as these are linked to oxidative stress. Despite the fact that the mechanisms linking oxidative damage to adipose tissue dysfunction remain largely unknown, we suggest that the MDA values determined in this study could reflect the degree of adipose oxidative stress [50]. Moreover, in agreement with our results, an increasing body of evidence supports the notion that diets high in glucose and fats, as occurs in obesity, may activate inflammatory signalling pathways in cells, potentially by increasing oxidative stress [51,52,53,54].

In recent years, it has been repeatedly reported that the gut microbiota in obese humans is different to that of lean people, both in terms of diversity and in the relative abundance of the dominant phyla *Bacteroidetes* and *Firmicutes* [55,56,57,58,59,60]. The different bacterial populations assessed in this study (*Akkermansia, Bacteroides-Prevotella-Porphyromonas, Bifidobacterium, Clostridia* cluster XIVa group (*Lactobacillus* group and *Faecalibacterium prausnitzzi*) represent more than 95% of the overall phylogenetic types of the human intestinal microbiota [8]. Our data identify the microorganisms related to *Lactobacillus* as a potential risk factor related with obesity. This result, that would have been surprising a few years ago, is in good agreement with some recent studies reporting higher levels of *Lactobacillus* in obese children [56] and adults [61]. Despite not reaching statistical significance, our results show a trend towards reduced levels of the *Bacteroides*-group in obese subjects. These observations are in good agreement with the increased Firmicutes/Bacteroidetes ratio [12] and the reduced *Bacteroides* levels repeatedly reported in obese subjects [57,60]. In addition, we found the majority of intestinal SCFA (acetate) to be another risk factor, with its levels increasing with BMI, which is in agreement with the higher levels of SCFA observed in obese subjects by other authors [15]. At this point, and based on the ability of the intestinal microbiota to act as an endocrine organ [50,56] we wonder to what extent this microbial ecosystem could be acting as a link between diet, systemic inflammation, metabolic dysfunction and obesity [62,63].

In this scenario, the traditional statistical analysis which considers the variables independently may be unrealistic when applied to complex biological systems; such is the case with obesity, in which there is a wide range of interrelated variables and mutually supporting disturbances. Therefore, we have gone one step further by evaluating the differences in the microbiota and dietary levels in those individuals in which different obesity-related factors occur together. The analyses of these obesity-related factors separated the individuals into two clusters. One of them (Cluster II) comprised the obese/over-weight participants showing higher serum concentrations of glucose, MDA and CRP. Interestingly, the negative association between *Bacteroides* and obesity reported previously by other authors [57,60,64] was also identified in our study when individuals were classified in clusters, while the association of *Lactobacillus* with obesity (BMI ≥ 30) did not raise statistical significance when also considering oxidative stress and inflammation related parameters. 

Both obesity and dietary patterns have been shown to be related to changes in gut microbiota composition [65,66]. Therefore, when analyzing the differences in diets between clusters I and II, it was not surprising that the higher intake of fat and oils and sweetened foods, suggestive of consuming a highly palatable diet, was associated with the presence of a chronic pro-inflammatory and pro-oxidant status linked to overweight and obese subjects from cluster II [67,68,69,70,71,72,73]. The mechanisms by which dietary components may produce changes at the microbiota level remain largely unknown. However, considering both our results and the evidence from experimental studies with germ free animals receiving a diet high in fats and sucrose, it is likely that the impact of these foods in obesity and related pathologies is mediated through microbiota modulation [74]. This is supported by the fact that we found an inverse association between the levels of MDA and those of the *Bacteroides* group.

In addition, the reduced intake of fruits, which have a known anti-obesogenic effect, could be also linked to changes in the microbiota composition. Vegetables and fruits were the major dietary sources of pectin in our cohort [23] which can be metabolized in the colon by bacteria such as *Bacteroides* resulting in the production of SCFA [75] that may exert different beneficial effects on the host [76]. Moreover, in our studied population, vegetables and fruits were also the main sources of vitamin C and carotenoids, compounds that have a known anti-inflammatory activity and also provide other bioactive compounds, such as polyphenols, that are involved in the regulation of some metabolic conditions linked to obesity [77,78] and inflammation [78]. Considering these preliminary results, we hypothesize that the *Bacteroides* group could act as a mediator in antioxidant metabolism, playing a key role in some of the health effects attributed to these compounds.

The limited sample size of this study may have somewhat hampered our ability to detect other significant associations. Moreover, although FFQ is the most suitable tool to describe long-term habits, its ability to accurately quantify the dietary intake is limited. In addition, fecal SCFA measurements comprise only the 5% to 10% that is not absorbed in the colon [75]. These facts underline the difficulty of drawing firm conclusions from the studies conducted in this area of research.

## Figures and Tables

**Table 1:** Bacterial groups, standard cultures, primers, and annealing temperatures (Tm) used for qPCR in this study.

Microbial Target	Strain Used for Standard Curve	Primer Sequence 5′–3′	Tm (°C)	Reference
*Akkermansia*	*Akkermansia muciniphila* CIP 107961	F: CAGCACGTGAAGGTGGGGAC	60	[32]
		R: CCTTGCGGTTGGCTTCAGAT		
*Bacteroides group* *Bacteroides-Prevotella-Porphiromonas*	*Bacteroides thetaiotaomicron* DSMZ 2079	F: GAGAGGAAGGTCCCCCAC	60	[32]
		R: CGCKACTTGGCTGGTTCAG		
*Bifidobacterium*	*Bifidobacterium longum* NCIMB 8809	F:GATTCTGGCTCAGGATGAACGC	60	[32]
		R: CTGATAGGACGCGACCCCAT		
*Faecalibacterium*	*Faecalibacterium prausnitzi* DSMZ 17677	F:GGAGGAAGAAGGTCTTCGG	60	[33]
		R: AATTCCGCCTACCTCTGCACT		
*Clostridia* XIVa *Blautia coccoides—Eubacterium rectale group*	*Blautia coccoides* DSMZ 935	F: CGGTACCTGACTAAGAAGC	55	[32]
		R: AGTTTYATTCTTGCGAACG		
*Lactobacillus group*	*Lactobacillus gasseri* IPLA IF7/5	F: AGCAGTAGGGAATCTTCCA	60	[32]
		R: CATGGAGTTCCACTGTCCTC		

**Table 2:** General characteristics of the studied population according to BMI.

	Normal Weight BMI ≤ 25.0 *n* = 20	Over Weight BMI 25.0–30.0 *n* = 35	*p*	Obesity BMI ≥ 30.0 *n* = 13	*p*
Age (years) ^a^	56.4 ± 10.1	51.7 ± 11.7	0.152	47.8 ± 10.2	0.033
Female (%)	80.0	51.4	0.036	53.8	0.110
BMI (kg/m^2^) ^a^	23.0 ± 1.5	27.5 ± 1.4	<0.001	34.1 ± 2.7	<0.001
Energy intake (Kcal/day) ^a^	1958 ± 537	1790 ± 482	0.261	2040 ± 548	0.681
Basal Metabolic rate (Kcal/day) ^a^	1280 ± 167	1416 ± 228	<0.001	1548 ± 324	<0.001
Sedentary lifestyle (%)	20.0	17.1	0.792	30.8	0.481
Current smokers (%)	27.8	26.5	0.729	25.0	0.978
Alcohol consumers (%)	70.0	54.3	0.252	61.5	0.614
Body fat (%) ^a^	26.2 ± 7.5	35.6 ± 9.3	<0.001	51.7 ± 10.8	<0.001
Blood parameters					
Serum glucose (mg/dL) ^a^	97.1 ± 14.2	96.0 ± 9.1	0.711	108 ± 11.1	0.020
Triglycerides (mg/dL) ^a^	100 ± 47.8	117 ± 56.9	0.288	147 ± 91.0	0.070
LDL/HDL ratio ^a^	2.5 ± 0.8	2.6 ± 0.8	0.869	2.3 ± 0.6	0.347
Leptin (ng/mL) ^a^	6.1 ± 4.3	9.2 ± 5.2	0.021	14.7 ± 6.8	<0.001
MDA (μM) ^a^	2.1 ± 0.6	2.2 ± 0.9	0.700	3.2 ± 1.6	0.012
CRP (mg/L) ^a^	0.9 ± 0.8	3.8 ± 8.4	0.150	5.4 ± 7.4	0.009

^a^ Results from univariate analysis were adjusted by gender and presented as mean ± standard deviation. Differences in categorical variables were examined using chi-squared analysis and presented as percentage (%). BMI, body mass index. LDL, low-density lipoprotein. HDL, high-density lipoprotein. MDA, malondialdehyde. CRP, C reactive protein. *p* value was calculated using normal weight volunteers as reference.

**Table 3:** Differences in fecal short chain fatty acids (SCFA) concentration and major microbial groups according to BMI (kg/m^2^) and results of linear regression analyses to estimate their association with BMI.

	Normal Weight BMI ≤ 25.0 *n* = 20	Over Weight BMI 25.0–30.0 *n* = 31	Obesity BMI ≥ 30.0 *n* = 13	BMI		
				*R*^2^	β	*p*
Model 1. Fecal SCFA concentration (mM)						
Acetate	35.7 ± 14.6	38.0 ± 16.8	46.6 ± 17.0	0.081	0.282	0.025
Propionate	14.4 ± 6.5	13.7 ± 6.7	17.1 ± 8.2	0.022	0.136	0.288
Butyrate	11.6 ± 8.7	10.3 ± 6.4	12.3 ± 9.0	0.047	0.040	0.748
Model 2. Microbial target(log nº cells/gram of faeces)						
*Akkermansia*	6.3 ± 2.2	5.6 ± 1.6	5.6 ± 2.1	0.026	−0.143	0.264
*Bacteroides-Prevotella-Porphyromonas*	8.8 ± 1.3	8.9 ± 1.1	8.2 ± 1.2	0.067	−0.245	0.052
*Bifidobacterium*	7.7 ± 2.0	8.2 ± 0.8	8.2 ± 0.7	0.090	0.126	0.305
*Clostridia* cluster XIVa group	7.7 ± 1.7	8.2 ± 1.3	8.4 ± 1.1	0.048	0.150	0.236
*Lactobacillus* group	5.7 ± 1.3	6.0 ± 1.1	6.7 ± 0.9 *	0.194	0.256	0.029
*Faecalibacterium prausnitzii*	7.3 ± 1.0	7.5 ± 1.0	7.7 ± 0.9	0.024	0.152	0.233

Results derived from multivariate analysis are presented as mean ± standard deviation. Variables included in model 1: acetate, propionate, butyrate and energy; model 2: *Akkermansia*, *Bacteroides-Prevotella-Porphyromonas*, *Bifidobacterium*, *Clostridia* cluster XIVa, *Lactobacillus* group, *Faecalibacterium prausnitzii* and energy. Linear regression analyses are adjusted by energy; *R*^2^, coefficient of multiple determination; β, standardized regression coefficient. * *p* ≤ 0.05.

**Table 4:** Differences in the parameters used for cluster analyses.

	Cluster I *n* = 38	Cluster II *n* = 26	*p*
BMI (kg/m^2^)	25.2 ± 2.7	30.3 ± 4.3	<0.001
Body fat (%)	30.5 ± 8.0	42.3 ± 13.3	<0.001
Blood parameters			
Serum glucose (mg/dL)	92.7 ± 8.2	108.2 ± 10.9	<0.001
LDL/HDL ratio	2.6 ± 0.8	2.5 ± 0.8	0.921
Leptin (ng/mL)	8.2 ± 5.1	10.8 ± 6.4	0.074
MDA (μM)	2.0 ± 0.6	2.8 ± 1.3	0.001
CRP (mg/L)	0.9 ± 0.9	6.7 ± 10.4	0.001

Univariate analysis was adjusted by gender and presented as mean ± standard deviation. LDL, low-density lipoprotein. HDL, high-density lipoprotein. MDA, malondialdehyde. CRP, C reactive protein.

**Table 5:** Differences in fecal SCFA and major microbial groups between clusters.

	Cluster I *n* = 37	Cluster II *n* = 24
Model 1. Fecal SCFA concentration (mM)		
Acetate	35.8 ± 14.8	44.8 ± 17.7 *
Propionate	14.0 ± 6.7	16.2 ± 7.3
Butyrate	11.1 ± 8.0	11.8 ± 7.7
Model 2. Microbial target (log nº cells/gram of feces)		
*Akkermansia*	6.0 ± 1.8	5.6 ± 2.2
*Bacteroides-Prevotella-Porphyromonas*	9.0 ± 1.0	8.3 ± 1.3 *
*Bifidobacterium*	8.1 ± 0.9	7.9 ± 1.8
*Clostridia* cluster XIVa group	7.9 ± 1.4	8.2 ± 1.4
*Lactobacillus* group	6.0 ± 1.1	6.1 ± 1.3
*Faecalibacterium prausnitzii*	7.4 ± 0.9	7.5 ± 0.9

Results derived from multivariate analysis are presented as mean ± standard deviation. Variables included in model 1: acetate, propionate, butyrate and energy; model 2: *Akkermansia, Bacteroides-Prevotella-Porphyromonas, Bifidobacterium, Clostridia* cluster XIVa, *Lactobacillus* group, *Faecalibacterium prausnitzii* and energy. * *p* ≤ 0.05.

**Table 6:** Differences in the intake of the major dietary antioxidants between clusters.

	Cluster I *n* = 38	Cluster II *n* = 26	*p*
ORAC, hydrophilic (μmol TE/day)	10367 ± 6641	6089 ± 4179	0.004
ORAC, lipophilic (μmol TE/day)	244 ± 193	138 ± 103	0.011
ORAC, Total (μmol TE/day)	10609 ± 6790	6229 ± 4224	0.004
Selenium (μg/day)	123 ± 40.3	115 ± 43.6	0.250
Total carotenoids (μg/day)	2391 ± 1538	1660 ± 1001	0.034
γ-Tocopherol (mg/day)	2.6 ± 1.1	2.1 ± 2.2	0.249
Vitamin C (mg/day)	222 ± 196	131 ± 102	0.021
Vitamin E (mg/day)	10.1 ± 4.1	12.7 ± 8.2	0.063
Total polyphenols (mg/day)	2057 ± 1076	1553 ± 975	0.043
Total flavonoids (mg/day)	435 ± 291	303 ± 232	0.049
Total phenolics (mg/day)	198 ± 192	224 ± 236	0.626
Flavanols (mg/day)	222 ± 187	189 ± 194	0.505

Results adjusted by energy derived from multivariate analysis are presented as mean ± standard deviation. ORAC, Oxygen radical absorbance capacity. TE, Trolox equivalents.
